# Social Capital–Accrual, Escape-From-Self, and Time-Displacement Effects of Internet Use During the COVID-19 Stay-at-Home Period: Prospective, Quantitative Survey Study

**DOI:** 10.2196/22740

**Published:** 2020-12-24

**Authors:** Cecilia Cheng, Yan-Ching Lau, Jeremy W Luk

**Affiliations:** 1 Social and Health Psychology Laboratory Department of Psychology The University of Hong Kong Hong Kong Hong Kong; 2 Division of Psychology and Language Sciences University College London London United Kingdom; 3 National Institute on Alcohol Abuse and Alcoholism Bethesda, MD United States

**Keywords:** coping, coronavirus, COVID-19, cyberaggression, cybervictimization, epidemic, gaming, mental health, psychological well-being, social networking, social support

## Abstract

**Background:**

COVID-19 has spread like wildfire across the globe, prompting many governments to impose unprecedented stay-at-home orders to limit its transmission. During an extended stay-at-home period, individuals may engage in more online leisure activities. Internet use is a double-edged sword that may have both desirable and undesirable effects on psychological well-being, and this study sought to disentangle adaptive from maladaptive internet use amidst this unusual health crisis.

**Objective:**

The objectives of this study were to assess the prevalence of probable depression during the COVID-19 stay-at-home period and to test three hypothesized risk reduction or risk elevation mechanisms, namely social capital–accrual, escape-from-self, and time-displacement effects.

**Methods:**

This study took place from March to May 2020 at the early stage of the pandemic. The study adopted a prospective design, with an online survey administered to 573 UK and 474 US adult residents at two assessment points 2 months apart.

**Results:**

The prevalence of moderate to severe depression was 36% (bootstrap bias-corrected and accelerated [BCa] 95% CI 33%-39%) at Time 1 (ie, initial time point) and 27% (bootstrap BCa 95% CI 25%-30%) at Time 2 (ie, follow-up time point). The results supported the social capital–accrual hypothesis by showing that the approach coping style was inversely associated with Time 2 depression through its positive associations with both social networking and perceived family support. The results also supported the escape-from-self hypothesis by revealing that the avoidant coping style was positively associated with Time 2 depression through its positive associations with both gaming and cyberbullying victimization, but the serial mediation model was no longer significant after Time 1 depression and some demographic risk factors had been controlled for. Finally, the results supported the time-displacement hypothesis by showing that gaming was positively associated with Time 2 depression through its inverse associations with social networking and perceived family support.

**Conclusions:**

During the extended stay-at-home period in the early stages of the COVID-19 pandemic, the prevalence of probable depression during the 2-month study period was high among the UK and US residents. Individuals with distinct coping styles may engage in different types of online leisure activities and perceive varying levels of social support, which are associated with risks of probable depression.

## Introduction

### Background Context

COVID-19 is an infectious disease caused by a novel strain of coronavirus known as SARS-CoV-2, which is deadly and highly transmissible [1,2]. Since the World Health Organization (WHO) reported the initial batch of confirmed COVID-19 cases on January 11, 2020, the disease has affected more than 68 million people and caused more than 1.6 million deaths in 191 countries and regions, globally, as of December 9, 2020 [3].

In response to the massive threat posed by COVID-19, numerous governments around the world have imposed a series of community-control measures in an attempt to curb its rapid transmission. For instance, the UK government implemented self-quarantine orders and school closures in March 2020 that lasted for around two months. During the same period, the majority of US states imposed similar stay-at-home orders. The residents of both countries were urged to avoid social gatherings and to stay at home, although they were allowed to go out for essential errands, such as buying groceries and attending medical appointments [4]. A US household survey revealed that the prevalence of probable depression during the early stage of the pandemic was 3 times higher than the prepandemic rate [5]. Systematic and meta-analytic reviews similarly showed that probable depression was prevalent (overall effect size of 34%, 95% CI 28%-41%) across the globe during the early stage of the pandemic [6,7] and that such alarming rates were 3-fold higher than the lifetime prevalence of depression from 1994 to 2014 (11%) [8]. Besides, sleep disturbance was also prevalent among the general public during the pandemic [9,10]. Apart from demographic factors such as gender and age, it is noteworthy that frequent social networking site (SNS) use was identified as a risk factor that heightened mental health problems [6,10].

### Internet Use and Mental Health Issues

This study examined major online leisure activities that people engaged in during the stay-at-home period and, more importantly, the problems related to such activities. With rapid advances in information and communication technology and the emergence of affordable mobile devices (eg, smartphones and tablets), people are increasingly reliant on the internet for social networking, entertainment, information, and online purchases. Among the array of online leisure activities, social networking is the most popular, as reflected in the escalation of the number of SNS users over the past two decades [11]. Facebook, the most widely used SNS, has more than 2.7 billion active users worldwide as of August 2020 [12]. Another highly popular online leisure activity is gaming; there were more than 2.3 billion active video gamers all around the world in 2019 [13].

The use of the internet can be beneficial, allowing connections with other people without geographical or time constraints [14]. Support from social network members rendered online has been reported to alleviate depressive symptoms [15,16]. However, certain problems brought about by internet use also merit attention [17]. For instance, internet use may increase the likelihood of cybervictimization, such as impersonation and social exclusion, which in turn increases the risk of depression [18,19]. Moreover, excessive engagement in online games may disrupt daily functioning [20,21]. Internet use is, thus, a double-edged sword that can enhance and compromise mental health.

### Risk Reduction and Elevation Mechanisms Underlying Internet Use

**Figure 1:**
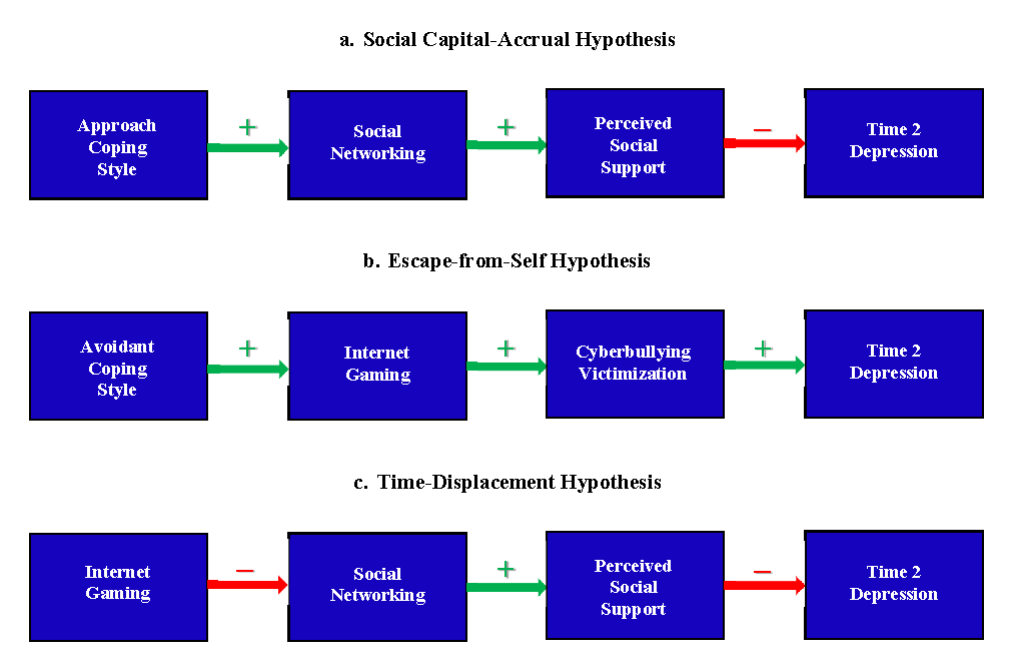
Conceptual models summarizing three hypotheses tested in this study. Time 2 is the follow-up time point.

This study aimed at unveiling both risk reduction and risk elevation mechanisms underlying leisure-time internet use and depression during the COVID-19 pandemic. Figure 1 depicts the conceptual models that illustrate three hypothesized mechanisms. According to the conservation of resources theory [22,23], individuals strive to obtain and retain resources that facilitate goal attainment, and they feel distressed when such resources are lost or unavailable after making significant effort. Since the onset of the ongoing pandemic, the social resources of many people had been severely depleted while the stay-at-home orders were in place. As face-to-face interactions were not feasible, people attempted to regain their reduced social resources through the internet. SNS has emerged as a popular venue for rendering and gaining social support in the cyber era [15,24], and users’ social support and subjective well-being were found to be increased after social networking [25,26].

A meta-analysis revealed considerable individual differences in the beneficial role of social networking in facilitating the accrual of online social capital [27]. Specifically, the meta-analytic findings showed that individuals who are motivated to use SNS to maintain contacts with members of their existing social networks tend to accrue more social capital that mitigates depressive symptoms. In a stressful encounter, an individual’s coping style plays a pivotal role in influencing the display of cognitive and behavioral responses, which, in turn, have mental health implications [28,29]. In the coping literature, a broad conceptualization has been widely adopted to dichotomize these psychological responses into two general coping styles: approach versus avoidant [29,30]. Approach coping refers to a tendency to undertake direct actions as an attempt to confront or change a stressful event, whereas avoidant coping refers to a tendency to retreat or divert attention away from a stressful event [31,32]. As individuals characterized by an approach coping style tend to face the stressful encounter and take proactive actions to tackle problems [33,34], we predict that these individuals may actively utilize SNS to accrue more support from members of their social network and may, thus, be less vulnerable to depression (see Figure 1a).

The meta-analytic findings further revealed that such mental health benefits, however, were absent among those who were motivated to use the internet as a refuge to avoid facing problems in the real world. According to the escaping-the-self theory [35], individuals tend to feel distressed when they realize that real-life outcomes are falling short of their expectations; they are motivated to evade such psychological discomfort by engaging in avoidant behaviors to divert their attention from existing problems. Their avoidant responses may elicit greater distress in the long run because the real-life problems remain intact [36,37]. Previous studies have consistently shown escapism as a major motivation of gaming that elicits adverse outcomes, such as cyberbullying victimization [38]. In the digital age, cyberbullying victimization is prevalent [39,40], and frequent such experiences enhance mental health risks [41,42]. In light of these theories and findings, we predict that individuals characterized by an avoidant coping style will tend to rely on gaming in an attempt to handle stress; however, their gaming behavior may increase their exposure to cyberbullying victimization, which may, in turn, enhance their susceptibility to depression (see Figure 1b).

It is noteworthy that investing more leisure time in gaming may reduce time spent social networking and, thus, the opportunities of reaping support from social network members. This notion stems from displacement theory [43], whose underlying assumption is that the total time spent on daily activities is constant. Hence, spending more time on a particular online activity results in less time on another. In light of the time-displacement theory, we predict that more time spent on gaming may be associated with less time spent on social networking and lower levels of social support, thus enhancing the risk of depression (see Figure 1c).

### Study Setting and Aims

This study was conducted at an early stage of the COVID-19 pandemic, when stay-at-home orders were in effect in many countries, including the United Kingdom and the United States. The study aimed at assessing the prevalence of probable depression and testing three hypothesized mechanisms that would reduce or enhance risks of depression: social capital–accrual, escape-from-self, and time-displacement effects (see Figure 1).

## Methods

### Study Design and Settings

A prospective design with a quantitative approach was adopted in this study, which comprised two assessment points. Initial (Time 1) data collection took place from March 16 to 22, 2020, the week after the WHO declared the COVID-19 outbreak a global pandemic and stay-at-home orders were first implemented in various countries. Follow-up (Time 2) data collection then took place from May 18 to 24, 2020, during which stay-at-home orders were gradually lifted in some regions of the United Kingdom and the United States.

### Study Sample and Eligibility Criteria

The participants were recruited through Prolific [44], a crowdsourcing platform for participant recruitment. Studies have shown that data generated via the crowdsourcing method were as reliable and valid as those obtained by offline methods, with participants reporting that they felt more comfortable sharing their personal data in crowdsourcing research than with other types of research [45,46]. Prolific was chosen because its participants were reported to have the most diverse demographic characteristics and to generate the highest data quality among popular crowdsourcing platforms [47,48].

Eligible participants were adults between the ages of 18 and 65 years who were living in the United Kingdom or the United States at the time of the study. Participants from other countries, those who did not take part in the follow-up assessment, and those who did not give informed consent were excluded. Data were collected from 1086 eligible participants, but 16 of them did not submit the survey, while 23 were timed out before they completed or submitted the survey (completion rate 96.4%). The final sample contained 1047 community adults.

### Measures

#### Overview

The online survey contained a set of validated measures, all of which were chosen because they were short and designed for use in surveys administered in general populations. All of the measures were administered at Time 1; the measure assessing depression was administered at both time points. The measures were arranged in a randomized order to counterbalance potential order effects. Participants were prompted if there were missing responses.

#### Coping Style

Both approach and avoidant coping styles were measured by the Coping Strategies Inventory–Short Form [49]. Each of the coping subscales included four items, each of which was rated on a 5-point scale, ranging from 1 (*never*) to 5 (*almost always*). A higher composite score (Cronbach α=.79 for the approach coping style subscale and .89 for the avoidant coping style subscale) indicates the endorsement of a particular coping style.

#### Cyberbullying Victimization

Cyberbullying victimization was assessed by the cybervictimization subscale of the Cyber-Aggression and Cyber-Victimization Scale [50]. The subscale contained eight items, each of which was rated on a 5-point scale, ranging from 1 (*never*) to 5 (*very often*). A composite score (Cronbach α=.88 in this sample) was derived by aggregating the scores for all items, with a higher score representing a greater degree of cyberbullying victimization.

#### Perceived Social Support

Perceived family support and friend support were measured by the family and friend subscales of the Multidimensional Scale of Perceived Social Support [51]. Each subscale had four items, each of which was rated on a 7-point scale, ranging from 1 (*very strongly disagree*) to 7 (*very strongly agree*). A subscale score (Cronbach α=.94 for the family subscale and .95 for the friend subscale) was derived from the sum of all item scores, with a higher score representing the subjective appraisals of greater perceived support from a particular social group.

#### Depression

Each participant’s level of depression was assessed by the Center for Epidemiological Studies–Depression Scale [52]. This depression scale comprised 20 items, each of which was rated on a 4-point scale, ranging from 0 (*rarely or none of the time*) to 3 (*all of the time*). A total depressive symptom score (Cronbach α=.84 and .80 at Time 1 and Time 2, respectively) was computed by summing all of the item scores, with a higher score representing a greater frequency and severity of depression. The conventional cutoff scores were 16 and over for mild depression and 23 and over for moderate to severe depression [52].

#### Time Allocated to Social Networking and Internet Gaming During the COVID-19 Pandemic

The participants reported the amount of leisure time (hours) they had dedicated on a typical day within the past week to two highly popular internet activities: social networking and gaming. Both of these items were found valid for assessing these online leisure activities [53,54].

#### Demographic Variables

At the end of the survey, the participants were asked to provide the following demographic information: age, sex, educational qualifications (ie, undergraduate degree holder or not), employment status (ie, employed, no need to work [ie, student, homemaker, or retired], or unemployed), marital status (ie, married or partnered or not), and ethnicity (ie, White or non-White). For conducting statistical analyses, the three-level employment status was recoded as a pair of dummy variables: employed (ie, 1=employed, 0=no need to work, 0=unemployed) and unemployed (ie, 0=employed, 0=no need to work, 1=unemployed). Age was recorded as a continuous variable, whereas all of the other demographic variables were dummy coded.

### Study Procedures

Survey invitations were sent by Prolific to its members who met the demographic criteria. The survey was distributed through the Qualtrics survey system. Participation was entirely voluntary. Potential participants were told the survey aim and length, payment rate for completion (ie, £7.80 [US $10.50] per hour), and the ethical approval number and agent. They were also assured that all data collected would be anonymous and kept in strict confidentiality in the investigators’ laboratory. All of the participants were requested to give informed consent prior to their participation at both time points, and were paid upon the completion of each survey. The data collected at the two time points were matched according to a unique code assigned to each participant by the survey platform.

### Strategy of Analysis

All statistical analyses were performed with SPSS, version 26 (IBM Corp). Independent-samples *t* tests were conducted to examine whether there were any between-country differences (ie, United Kingdom vs United States) in the study variable. To test the three hypothesized mechanisms underlying the experience of Time 2 depression during the early stage of the COVID-19 pandemic, serial mediation analysis was performed using Model 6 of the SPSS macro PROCESS, version 3.5 [55], which used ordinary least squares procedures to estimate the hypothesized effects. Three sets of analyses were performed. First, initial tests for all three hypothesized serial mediation effects were conducted without any covariates. Second, the hypothesized effects were then tested with Time 1 depression as a covariate in order to capture temporal changes in depression level. Third, both Time 1 depression and relevant demographic variables were included as covariates to rule out any additional confounding factors. Recent reviews on depression assessed during the COVID-19 pandemic revealed some demographic characteristics (ie, age, gender, country, and finance-related factors) as risk factors that enhanced susceptibility to depression [5,6]. In addition, marital status was identified to be crucial for social connectedness [56]. Taken together, the following demographic risk factors were included as covariates in the third set of model testing: age; gender; country; employment status, which was represented by a pair of dummy variables (ie, employed or unemployed); and marital status.

In all of the mediation analyses, the scores on the predictor variables were centered to reduce multicollinearity [57]. Bootstrapping was conducted with 10,000 iterations (2-tailed significance), with the bootstrap bias-corrected and accelerated (BCa) interval employed to estimate the CIs. The hypothesized effects were deemed statistically significant if zero was excluded in the bootstrapped CIs.

### Ethical Considerations

The research protocol was reviewed and approved by the human research ethics committee of the University of Hong Kong (approval No. EA2002033) prior to Time 1 data collection. All study procedures were carried out in accordance with the ethical rules of the Declaration of Helsinki of 1975, as revised in 2008.

## Results

### Sample Characteristics

The sample from this study consisted of 1047 adults residing in the United Kingdom and the United States at the time of the study. Participants from the two countries did not differ significantly in terms of the study variables (*P* values were above .14), except for Time 1 depression (t_1043_=11.91, *P*<.001). The UK participants (mean score 22.32, SD 13.24) reported higher levels of depression at Time 1 than their US counterparts (mean score 18.11, SD 11.08). The two groups were pooled to enhance statistical power, but the demographic variable of country was included as a covariate in model testing.

The pooled sample of 1047 participants comprised 481 men (45.9%) and 563 women (53.8%), as well as 3 participants (0.3%) who did not indicate their sex. The average age was 44.10 years (SD 12.59). Slightly more than half of the participants were undergraduate degree holders or above (596/1044, 57.1%) and were married or partnered (569/1036, 54.9%). Most participants were ethnically White (887/1041, 85.2%).

On average, the participants spent 1.35 hours (bootstrap BCa 95% CI 1.28-1.42) on social networking and 1.70 hours (bootstrap BCa 95% CI 1.59-1.81) on gaming.

### Prevalence of Probable Depression at the Early Stage of the COVID-19 Pandemic

**Figure 2:**
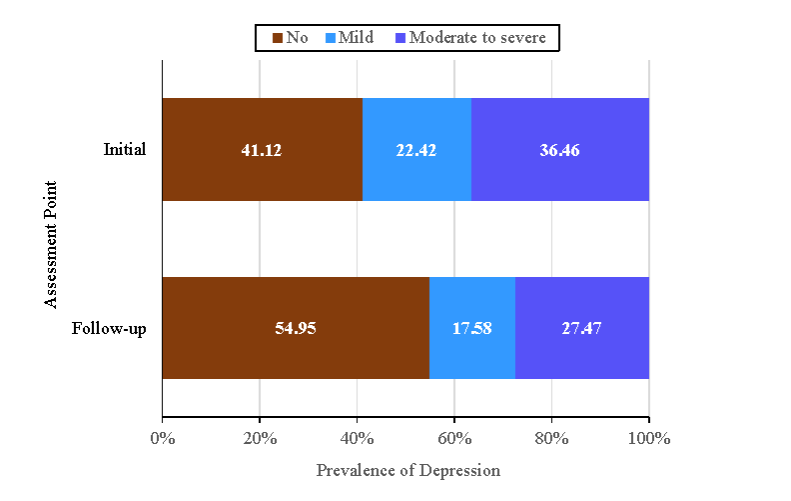
Prevalence of depression at the initial (March 16-22, 2020) and follow-up (May 18-24, 2020) assessment points during the COVID-19 pandemic.

The average depression scores at the initial and follow-up time points were 20.41 (bootstrap BCa 95% CI 19.60-21.12) and 16.74 (bootstrap BCa 95% CI 16.06-17.42), respectively. Figure 2 illustrates the proportion of participants categorized as having no, mild, and moderate to severe depression. According to the standard cutoff scheme, the prevalence of moderate to severe depression was quite high: 36% (bootstrap BCa 95% CI 33%-39%) at Time 1 and 27% (bootstrap BCa 95% CI 25%-30%) at Time 2.

### Mediation Analyses Unveiling Risk Reduction and Elevation Mechanisms

The results of serial mediation analysis for testing the various hypotheses are summarized in Table 1. The various serial mediation models were first tested without any covariates entered (see the upper panel of Table 1). Consistent with the social capital–accrual hypothesis, the results showed that the indirect effects of the approach coping style on Time 2 depression via social networking and perceived social support—both family and peer support—were significant. In line with the escape-from-self hypothesis, the indirect effect of the avoidant coping style on Time 2 depression via gaming and cyberbullying was significant. Consistent with the time-displacement hypothesis, the indirect effects of gaming on Time 2 depression via social networking and perceived social support—both family and peer support—were significant.

A highly similar pattern of findings was obtained after controlling for Time 1 depression (see the middle panel of Table 1), except for the set of findings for testing the escape-from-self hypothesis. Specifically, this particular serial mediation model was no longer significant. We then performed post hoc tests for simple mediation effects (ie, PROCESS Model 4), and gaming was found to mediate the positive association between the avoidant coping style and Time 2 depression after the baseline depression level had been controlled for.

In the final set of models, Time 1 depression and some demographic risk factors of depression were included as covariates (see the lower panel of Table 1). Similar to the previous set of analyses, only the simple mediation effect of gaming between the avoidant coping style and Time 2 depression was significant. In addition, the two serial mediation effects with perceived peer support as a mediator became nonsignificant when the cluster of demographic risk factors had been entered. In summary, this final set of analyses showed strong support for both social capital–accrual and time-displacement hypotheses when perceived family support was a mediator.

**Table 1:** Summary of model testing for three hypotheses (N=1047).

Indirect effect		Effect	Bootstrap, SE	BCa 95% CI^a^	*R* ^2^
**Models with no covariates**					
	H1^b^a: APC^c^ **→** SNW^d^ **→** PFS^e^ **→** Time 2 depression	–0.0144	0.0055	–0.0268 to –0.0055	0.2000
	H1b: APC **→** SNW **→** PPS^f^ **→** Time 2 depression	–0.0171	0.0063	–0.0306 to –0.0063	0.2200
	H2^g^: AVC^h^ **→** Gaming **→** CBV^i^ **→** Time 2 depression	0.0199	0.0084	0.0064 to 0.0391	0.2800
	H3^j^a: Gaming **→** SNW **→** PFS **→** Time 2 depression	0.0431	0.0121	0.0227 to 0.0703	0.1700
	H3b: Gaming **→** SNW **→** PPS **→** Time 2 depression	0.0340	0.0102	0.0164 to 0.0565	0.1700
**Models with Time 1 depression as a covariate**					
	H1a: APC **→** SNW **→** PFS **→** Time 2 depression	–0.0129	0.0052	–0.0249 to –0.0046	0.2300
	H1b: APC **→** SNW **→** PPS **→** Time 2 depression	–0.0152	0.0059	–0.0279 to –0.0051	0.2500
	H2: AVC **→** Gaming **→** CBV **→** Time 2 depression^k^	0.0095	0.0065	–0.0003 to 0.0250	0.3000
	H2 (post hoc test): AVC **→** Gaming **→** Time 2 depression	0.0296	0.0145	0.0054 to 0.0619	0.2200
	H3a: Gaming **→** SNW **→** PFS **→** Time 2 depression	0.0375	0.0121	0.0174 to 0.0638	0.2000
	H3b: Gaming **→** SNW **→** PPS **→** Time 2 depression	0.0306	0.0104	0.0131 to 0.0539	0.2000
**Models with Time 1 depression and demographic risk factors as covariates^l^**					
	H1a: APC **→** SNW **→** PFS **→** Time 2 depression	–0.0037	0.0020	–0.0083 to –0.0008	0.5100
	H1b: APC **→** SNW **→** PPS **→** Time 2 depression^k^	–0.0017	0.0014	–0.0051 to 0.0005	0.5000
	H2: AVC **→** Gaming **→** CBV **→** Time 2 depression^k^	0.0053	0.0042	–0.0005 to 0.0156	0.5300
	H2 (post hoc test): AVC **→** Gaming **→** Time 2 depression	0.0182	0.0104	0.0019 to 0.0417	0.5000
	H3a: Gaming **→** SNW **→** PFS **→** Time 2 depression	0.0095	0.0044	0.0025 to 0.0197	0.5100
	H3b: Gaming **→** SNW **→** PPS **→** Time 2 depression^k^	0.0042	0.0028	–0.0002 to 0.0107	0.5000

^a^Bias-corrected and accelerated (BCa) bootstrapped CIs, computed based on 10,000 bootstrap samples, were employed to interpret the significance of results for indirect effects instead of inferential tests [55].

^b^H1: social capital–accrual hypothesis, with either perceived family support (a) or perceived peer support (b).

^c^APC: approach coping style.

^d^SNW: social networking.

^e^PFS: perceived family support.

^f^PPS: perceived peer support.

^g^H2: escape-from-self hypothesis.

^h^AVC: avoidant coping style.

^i^CBV: cyberbullying victimization.

^j^H3: time-displacement hypothesis, with either perceived family support (a) or perceived peer support (b).

^k^This model resulted in nonsignificant findings.

^l^Demographic risk factor covariates include gender, age, country, employment status (ie, employed or unemployed), and marital status.

## Discussion

### Principal Findings

This study was conducted at a time when stay-at-home orders were in effect during the early stage of the COVID-19 pandemic. The findings indicate a high prevalence of probable depression immediately after the WHO’s designation of the novel disease as a global pandemic. Specifically, more than one-third of our participants reported moderate to severe depression in the initial assessment period. Although the prevalence rate was lower at the second assessment that took place 2 months later, it remained at a relatively high level, with around one-quarter of the sample reporting moderate to severe depression at the follow-up period. These findings indicate that it was quite common for UK and US residents to experience some forms of depression when self-quarantining in their respective countries. The prevalence of probable depression obtained at both time points was about 3 times higher than the prepandemic prevalence [5]. Similarly, the prevalence of probable depression obtained at the initial time point was 3 times higher, and that obtained at the follow-up time point was about 2.5 times higher, than the lifetime prevalence of depression from 1994 to 2014 reported in a review [8].

Consistent with the social capital–accrual hypothesis, the findings show that individuals who report higher levels of approach coping tend to spend more leisure time on social networking and perceive a higher level of family support, which is related to lower subsequent levels of depression during the extended stay-at-home period. In contrast, individuals who report higher levels of avoidant coping tend to spend more time gaming, which is related to higher subsequent levels of depression. In line with the time-displacement hypothesis, gaming time is inversely associated with both social networking time and perceived family support, which is inversely associated with subsequent levels of depression.

### Recommendations

These findings highlight the mental health implications of leisure-time internet use during the extended stay-at-home period at the early stage of the COVID-19 pandemic. Clinicians should be aware of individual differences in coping styles and assess whether the amount and type of internet use may increase or decrease risk for depression. Online professional support may be helpful to maintain psychological well-being if support from one’s social network is unavailable or perceived to be scant during social isolation. If in-person appointments for mental health services cannot be made, the use of telemedicine can bridge this gap by strengthening adaptive coping with various types of stressors during the pandemic [58]. Further, the general public may benefit from increased awareness about mental health issues during the extended stay-at-home period and acquire practical strategies to reduce risk of depression, such as limiting sources of stress, social support seeking, and maintaining a regular routine [59].

As excessive leisure-time internet use, especially for gaming, can heighten risks of depression, clinicians are advised to expend more effort probing into the amount of leisure time their clients spend on the internet as part of a comprehensive mental health assessment, as well as to evaluate their clients’ engagement in specific leisure activities (eg, social networking and gaming) that are likely to enhance or mitigate depression risk [60]. For instance, limiting the amount of time devoted to unconfirmed or questionable sources of COVID-19–related information, as well as maintaining a routine that does not involve excessive internet use, could reduce depressive symptoms [59].

The identification of mechanistic pathways leading to psychological issues during the pandemic has been proposed as a research priority owing to its relevance to refining interventions [61]. This study indicates that depression experienced during the extended stay-at-home period is a function of three crucial factors: coping style, the type of leisure-time internet use, and the type of social support. The three pathways identified in our study have practical implications. Firstly, the social capital–accrual pathway highlights the importance of the approach coping style and social networking in an attempt to elicit greater social support from family members. Secondly, the escape-from-self pathway specifies the maladaptive role of the avoidant coping style and gaming. Finally, the time-displacement pathway indicates that more time spent on gaming may reduce time spent on social networking and perceptions of family support. Clinicians who deliver psychological interventions to clients who lack family support should evaluate their clients’ patterns of leisure-time internet use as a potential contributor to depression.

### Study Limitations and Future Research Directions

Prior to concluding, several study limitations and directions for future research should be noted. First, the participants in this study were residents of the United Kingdom or the United States, both with some of the highest internet penetration rates in the world [62]. Accordingly, our findings may not be applicable to other COVID-19 hard-hit countries, particularly those with low internet penetration rates. Moreover, the participants were all residents of individualist countries, whose cultural values differ considerably from those shared by members of collectivist countries, such as Brazil and India [63-65]. Studies have shown considerable cultural differences in the motivation of social networking [66] and in the prevalence of internet addiction and internet gaming disorder [67,68]. Although the prevalence rates of probable depression of our two samples were comparable to those reported in a systematic review during the COVID-19 pandemic in eight countries across four continents [6], future studies should expand the scope of our research by recruiting participants from an array of countries with varying levels of internet penetration and cultural values to allow for more extensive multinational and cross-cultural comparisons.

Second, the prospective study was conducted during an early stage of the COVID-19 pandemic when unprecedented stay-at-home orders were in place. It is noteworthy that the findings reflect the participants’ initial psychological responses to the pandemic and may, thus, not be generalizable to other waves of the pandemic or to the postpandemic period. Previous studies have shown social isolation for extended periods to have long-term undesirable psychological impacts [69,70], and mental health issues fluctuated drastically across various waves of a disease outbreak [71]. Follow-up studies with a longer time horizon should be conducted to examine the potential chronic impact once the current drastic control measures have been lifted.

Finally, our study focused on the two most popular online leisure activities, namely social networking and gaming, because validated measures are only available for assessing time spent on these two types of activities. Nowadays, individuals also browse the internet for other purposes (eg, watching videos and movies and shopping), and it is worthwhile to expand the scope of internet use if validated measures are available for tapping these activities as well. As interpersonal interactions are minimal in many of these alternative activities, the pattern of findings may differ from those yielded in this study. Also, studies have demonstrated that interpersonal behaviors tend to differ among players of distinct game genres (eg, single-player vs multiplayer and cooperative vs competitive gamers) [72-75], and the pattern of findings may vary according to the type of games played on a regular basis. Future research may benefit from a nuanced analysis that takes the game genre and type of internet activity into consideration to enhance explanatory and predictive utility.

### Conclusions

During the extended stay-at-home period mandated to deal with COVID-19 transmission, depression was found to be prevalent. The findings reported herein indicate that leisure-time internet use can reduce or increase risks of depression, depending largely on the coping style of the users. As increasing numbers of people worldwide are being required to stay at home, additional studies should be conducted to obtain a more nuanced picture of the impacts of coping style, engagement in online leisure activities, and family dynamics on mental health.

## Abbreviations

BCa: bias-corrected and accelerated
SNS: social networking site
WHO: World Health Organization
